# Dietary diversity and associated factors among children aged 6–23 months in Sinan *Woreda*, Northwest Ethiopia: a cross-sectional study

**DOI:** 10.1186/s40795-018-0214-2

**Published:** 2018-02-17

**Authors:** Habtamu Temesgen, Tebikew Yeneabat, Muluken Teshome

**Affiliations:** 1 Department of Nutrition and Food Science, College of Health Sciences, Debre Markos University, Debre Markos, Ethiopia; 2 Department of Midwifery, College Health Sciences, Debre Markos University, Debre Markos, Ethiopia; 3 Department of Public Health, College of Health Sciences, Debre Markos University, Debre Markos, Ethiopia

**Keywords:** Children aged 6–23 months, Dietary diversity, Sinan *Woreda*

## Abstract

**Background:**

Child malnutrition accounted by poor dietary diversity is common in developing countries contributing for child morbidity and mortality. It also has an impact on child growth and development. Almost all nutritional related problems are preventable by implementing infant and child feeding strategies. The first two years of life are particularly important to reverse the nutritional problems by achieving dietary diversity feeding.

The study aimed to assess dietary diversity and its associated factors among 6–23 months old children in Sinan *Woreda*, Northwest Ethiopia.

**Methods:**

We conducted community based cross-sectional study among children aged 6–23 months in Sinan *Woreda* from February 16 to March 10, 2016. Random sampling technique was used to select 740 samples. Data on children’s dietary diversity of the last 24 h were collected through interview of mothers. Data were entered into EpiData version 3.1 and analysis was performed using SPSS version 20. The bivariate and multivariable logistic regression analyses were done to identify the independent factors associated with sub-optimal dietary diversity among children aged 6–23 months.

**Results:**

Seven hundred thirty six samples were included in the analysis with the response rate of 99%. Optimum dietary diversity was observed in 13% children. The dominant food groups consumed were grains. Availability of media sources at household [Adjusted Odds Ratio (AOR) = 2.77 (1.65–4.68)], availability of cow milk in the household [AOR = 2.39 (1.31–4.35)], women’s involvement in decision-making at household level [AOR = 2.07 (1.02–4.20)], institutional delivery service utilization [AOR = 2.40 (1.24–4.67)], receiving assisted delivery service [AOR = 2.36 (1.12–4.98)], receiving postnatal care [AOR = 2.07 (1.18–3.63)], distance far from the health center [AOR = 3.11 (1.66–5.83)] and meal frequency being four and above [AOR = 3.31 (1.53–7.18)] were associated with dietary diversity.

**Conclusion:**

This study concluded that optimum dietary diversity among children aged 6–23 months in Sinan *Woreda* is low. Meal frequency is positively associated with dietary diversity. Women involvement at household decision making improves dietary diversity of children. Ensuring maternal health service utilization can contribute for better dietary diversity of children aged 6–23 months. Large scale an interventional based research has to be conducted.

## Background

Globally, malnutrition affects social and economic development of a country. It is one of the commonest causes of under-five mortality, accounting for 45% of the 5.9 million deaths of under-five children in 2015 [1]. Those who survived from chronic malnutrition were suffering from wasting, irreversible physical and mental development [2–7]. Infant and young child feeding is a key area to improve child survival and promote healthy growth and development. The first two years of a child’s life are particularly important, as optimal nutrition during this period lowers morbidity and mortality, reduces the risk of chronic disease, and fosters better development overall [3].

The Sustainable Development Goals (SDGs) aimed to ensure healthy lives and promote well-being for all children. The SDG 3 target 3.2 aimed to end preventable deaths of newborns and under five year children by 2030 [8]. United Nations has emphasized that implementations to achieve the SDGs should acknowledge the nutrition priorities in which all of the SDGs have relations with nutrition. Improvements in human nutrition represent both a maker and a marker of sustainable development [9].

Improvement in human nutrition can be achieved through dietary diversity. Dietary diversity scores of the individuals reflect nutrient adequacy. Hence, nutrient adequacy of the diet consumed by the individuals depends on the individual dietary diversity score [10, 11]. World Health Organization (WHO) has designed a diet quality indicator to assess child feeding practices among children 6–23 months old. Diversified diet helps children to have appropriate nutrients needed for adequate growth and development [12, 13].

World health organization recommends children to consume foods among the seven food groups [(1). Grains, roots and tubers; (2). Legumes and nuts; (3). Dairy products; (4). Flesh foods (meats/fish/poultry); (5). Eggs; (6). Vitamin A-rich fruits and vegetables; and (7). Other fruits and vegetables] [14]. Infant and Young child feeding (IYCF) practices recommend that breastfed children aged 6–23 months must fed four or more other food groups daily. Non-breastfed children should feed milk or milk products, in addition to four or more food groups [15]. Despite of the importance of diversified food for children aged 6–23 months, only less than a fourth of them globally get diversified food and had recommended meal frequency that are appropriate for their age [3].

Only 4% of breastfed children in Ethiopia have received diversified diet [15]. In Southwest Ethiopia, about 39% of the children were categorized in the low Dietary Diversity Score [16]. The national prevalence of under-five stunting in Ethiopia is 44% and it is 52% in Amhara [15].

Globally, studies show that different socio-demographic and economic characteristics of mothers/care takers and children are associated with dietary diversity for children aged less than two years [14, 17–24]. Place of residence, age of the child, maternal education, birth order, wealth index and number of children less than five years old in the household were some of the factors which determine minimum dietary diversity [11, 25].

The ministry of health of Ethiopia has targeted to improve nutritional practices for children of 6–23 months age. The first 23 months are critical period of opportunity for health and development of children. However, there is still low dietary diversity practice in children age 6–23 months according to 2011 Ethiopian Demographic and Health Survey (EDHS) report [15, 26]. There are no documented studies on the dietary diversity feeding and its associated factors among children aged 6–23 months in Sinan *Woreda*. This study aimed to identify the magnitude of low dietary diversity among children aged 6–23 months and associated factors.

## Methods

### Study design and setting

Community based cross-sectional study was conducted in Sinan *Woreda*, East Gojjam Zone from February 16 to March 10, 2016. Sinan *Woreda* is located at 327 km from Addis Ababa in Northwest and 303 km from Bahir Dar in Southeast. The estimated population of the *Woreda* in 2015/16 is about 114,475. The estimated number of the under-five year children is 15,499. The total number of children aged 6–23 months account 4315 (Sinan worea health office: Health sector woreda base strategic plan report of sinan woreda, unpublished). Quantitative data were collected from mothers.

We obtained ethical clearance from the Ethical review committee of College of Health Sciences of Debre Markos University. Verbal informed consent, which was prepared in written form and dictated to the respondents during data collection, was obtained from the study participants after explaining the purpose of the study and the benefits. Respondents were interviewed voluntarily, anonymously and confidentiality also was assured. All participants were allowed to ask questions throughout data collection and could refuse to answer questions or stop the interview at any moment.

### Sample size determination and sampling procedure

Sample size was determined using single population proportion formula. $N=\frac{{\left(Z\frac{a}{2}\right)}^2p\left(1-p\right)}{d^2}$ [27], where, **p** represents the proportion of children who took the optimum dietary diversity, which was 12.6% taken from the study done in Ethiopia [28]. To get the optimum sample size, 3% margin of error (d) was considered with 95% confidence interval.

$$ N=\frac{(1.96)^20.126\left(1-0.126\right)}{0.03^2}470 $$

We added 5% for non-response rate and multiplied by 1.5 because of design effect. Then the final sample size was 740.

Of the 17 administrative Kebeles in the Woreda, seven were selected randomly for the study. Proportional to population size allocation was done to select the desired samples from each selected Kebele. Sampling frame, based on community-based health information system of family folder in health posts, was constructed. Lists of all mothers having children age 6–23 months with Community Health Information System (CHIS) number in selected kebeles were used to select the respondents through computer generated methods of random sampling. Children aged 6–23 months who did not start complementary food were excluded from this study.

### Data collection procedure and measurements

Questionnaires were composed of dietary diversity score adapted from the World health organization IYCF guideline which contains seven food groups for young child (6 to 23 months old) dietary diversity (Grains, roots and tubers; Legumes and nuts; Dairy products (milk, yogurt, and cheese); Flesh foods (meat, fish, poultry and liver/organ meats); Eggs; vitamin-A rich fruits and vegetables; Other fruits and vegetables) [7] and maternal and child demographic characteristics adapted from EDHS 2011 [15]. Questionnaires were first prepared in English and translated into Amharic Version, which later on, were translated into English. Amharic version questionnaires were used to collect data. Pre-test was done on 5% of the sample, two weeks before the actual data collection at Machakel *Woreda*. Data on dietary diversity were collected through face to face interviewing of mothers having children aged 6–23 months by allowing them freely to recall the type of food items they feed to their child/children within the last 24 h. Twelve data collectors, recruited based on their previous experience, and two supervisors had participated in data collection process. One day training on questionnaires and methods of data collection procedures was provided to data collectors and supervisors. Supervisors had checked completeness and consistency of the collected data by reviewing each completed questionnaire daily.

The dependent variable is Dietary diversity (dichotomized as optimal /suboptimal). Independent variables include socioeconomic and demographic characteristics (like age, educational status, occupation etc.), health utilization, and child health characteristics.

#### Operational definition

**Optimal dietary diversity:** Dietary diversity was defined as optimal if children (aged 6–23 months) received foods from at least four of seven food groups [(1) Grains, roots, and tubers, (2) Legumes and nuts, (3) Dairy products, (4) Flesh foods, (5) Eggs, (6) Vitamin-A rich fruits and vegetables, (7) Other fruits and vegetables, within the preceding 24 h of interview.

**Sub-optimal dietary diversity:** was defined as receiving three foods or fewer within 24 h [7, 29].

The terms **‘Women and mothers’** are used interchangeably in this study.

### Statistical analysis

Data were entered into EpiData version 3.1 with double entry verification. Analysis was performed using SPSS version 20.0. Frequency and cross-tabulation were used to present descriptive data. Both the bivariate and multivariable logistic regression analyses were performed to assess the association between dependent and independent variables. Independent variables that showed *P* < 0.2 at 95% CI in the bivariate logistic regression analysis were included in multivariable logistic regression model. *P* < 0.05, with 95% CI, was considered to declare the variables significantly associated with the dependent variable.

## Results

Of the total 740 sampled mothers/care takers who had children 6–23 months, 736 of them participated in the study with the response rate of 99%.

### Socio-demographic characteristics of mothers

The mean (±SD) age of the mothers was 30 (±5.69) years. Majority (58%) were in the age group of 25–34 years. All of them belong to Orthodox Tewahido Christian in their religion and Amhara in their Ethnicity. More than 95% of them were married. More than three-fourth (78%) of them were unable to read and write. Majority (75%) of them were farmers. More than 92% of them were from male headed households and 82% mothers had involved in decision-making activities (Table 1).

**Table 1:** Socio-demographic and economic characteristics of mothers having child aged 6–23 months in Sinan *Woreda*, Northwest Ethiopia, 2016 (*N* = 736)

Variables		Frequency	
		N	%
Maternal education level	Unable to read and write	572	78
	Read and write only	92	13
	Primary school (1–8 grade)	22	3
	Secondary school (9–12 grade)	33	4
	Higher education	17	2
Father’s education	Unable to read and write	382	52
	Read and write only	239	32
	Primary school (1–8 grade)	54	7
	Secondary school(9-12grade)	41	6
	Higher education	20	3
Maternal Occupation	Housewife	102	14
	Government employed	20	3
	Merchant	54	7
	Farmer	549	75
	Others	11	1
Fathers’ occupation	Farmer	558	76
	Government employed	39	5
	Private employed	10	1
	Merchant	75	10
	Daily laborer	26	4
	Others	28	4
Household family size	< 4	190	26
	> = 4	546	74
Number of < 5 years children	1	611	83
	> = 2	125	17
Availability of Media sources^a^ at the households	Yes	155	21
Exposure to available media sources	Yes	139	90
Available farmland	Yes	562	76
Cultivating vegetables	Yes	194	26
Available livestock	Yes	579	79
Having cow milk and feed the child	Yes	98	13
Mother involved in decision-making	Yes	604	82

^a^Includes Radio and Television (TV)

### Characteristics of the children aged 6–23 months

The mean (±SD) age of the children was 14.21(±5.3) months. About half (52%) of them were male. Majority (75%) of them were second and above child for the interviewed mothers. Seventy two (10%) of them had experience of morbidity within the preceding two weeks. About 66% of them had received growth-monitoring service and 75% of them received measles vaccine (Table 2).

**Table 2:** Selected health related characteristics children aged 6–23 months in Sinan *Woreda*, Northwest Ethiopia, 2016 (*N* = 736)

Variables		Frequency	
		*N*	%
Child’s age in months	6–11	297	40
	12–17	215	29
	18–23	224	30
Birth order	First	182	25
	Second to fourth	385	52
	Fifth and above	169	23
Morbidity for the last two weeks	Yes	72	10
Type of morbidity	Diarrhea	24	33
	Fever	15	21
	Cough	24	33
	Others	9	13
BCG Vaccinated	Yes	723	98
Pentavalent1 vaccinated	Yes	724	98
Pentavalent3 vaccinated	Yes	710	96
Measles vaccinated	Yes	555	75
Growth monitoring practice follow up	Yes	489	66

*BCG* Bacillus Calmette-Guérin, *Pentavalent* A vaccine against Diphtheria, Pertusis, Tetanus, Hepatitis B and Haemophilus influenza type B

### Health utilization, feeding practice and behavioral characteristics of mothers

More than three-fourth (78%) of mothers were multigravida. Majority (85%) of them have used family planning before the last pregnancy and 90% of them had Antenatal Care (ANC) follow up during the last pregnancy. More than half (54%) of them gave birth to their last child at health institution. Health professionals assisted 53.3% deliveries. Three hundred forty (46%) mothers have received Postnatal care (PNC) following their last childbirth. Six hundred twenty five (85%) of them have received breast-feeding counseling. Majority (92%) of them knew when to start complementary feeding (CF) and 63% of them have initiated complementary feeding timely. Majority (98%) of them reported feeding breast milk to their children. More than one-third (40%) of them reported feeding their child three times per day (Table 3).

**Table 3:** Health service utilization, information on child feeding and feeding practice of mothers in Sinan *Woreda*, Northwest Ethiopia, 2016 (*n* = 736)

Variables		Frequency	
		*N*	%
Gravidity	Primigravida	165	22
	Multigravida	571	78
ANC follow up for the index child	Yes	661	90
Place of delivery	Home	310	42
	Health posts	30	4
	Health institutions	396	54
Mode of delivery	Spontaneous vaginal delivery	693	94
	Assisted deliveries	43	6
PNC follow up for the index child	Yes	340	46
Utilizations of Family planning before current pregnancy	Yes	627	85
Counseled on BF	Yes	625	85
Time to reach nearby health center	Less than 2 h	641	87
	2 h and above	95	13
Knew when to start CF	Yes	679	92
Initiation of CF	Timely initiate CF	463	63
	Not timely initiate CF	273	37
Currently breast feed	Yes	721	98
Source of information of CF	Health professionals	171	23
	family members	141	19
	HEWs	424	58
Meal frequency of children	1-2times/day	165	22
	Three	295	40
	Four and above	276	38
Feeding material	Feed by cup or spoon	459	62
	Active feeding	205	28
	Bottle feeding	25	3
	Others^a^	47	6
Consistency of the CF	Gruel	176	24
	Slightly dense porridge	192	26
	Dense porridge	34	5
	Usual household food	313	43
	Others^b^	21	3

^a^feeding by caretakers hands

^b^locally made bread, tea

### Dietary diversity and type of diversified food items

**Fig. 1:**
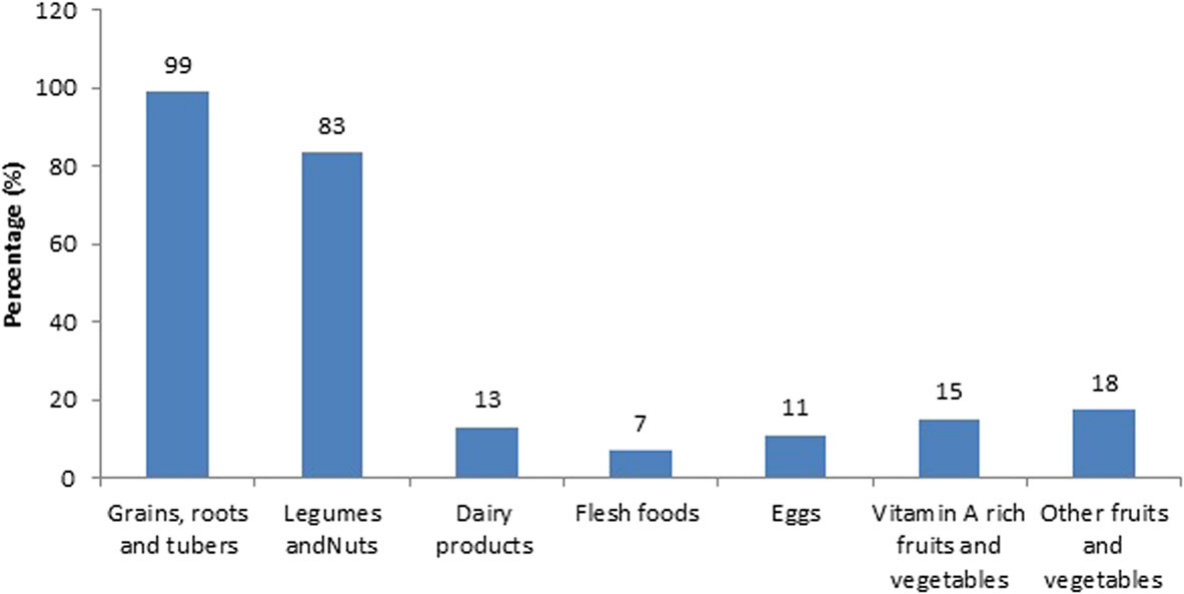
Types of dietary groups given to children aged 6–23 months in Sinan *Woreda*, Northwest Ethiopia, 2016

Children who fed four or more food items within 24 h preceding data collection were 13%. Of children included in the study, 74(10%), 379(52%) and 189(26%) consumed from one, two and three food items were respectively. The mean (±SD) dietary diversity score (DDS) was 2.45(±0.95). The dominant food groups consumed were grains (99%) followed by legumes (83%) (Fig. 1).

### Factors associated with dietary diversity among children aged 6–23 months

In multivariable logistic regression analysis, those mothers who had available media at the household level were 2.77 times more likely to feed their children with optimum dietary diversity compared to those who had no media sources. Those who had milk available at household were 2.38 times more likely to feed their children with optimum dietary diversity. Women who have been involved in decision-making were 2.07 times more likely to feed their children with optimum dietary diversity. Feeding with optimum dietary diversity to their children was 2.40 times more among mothers who have delivered their last child at health institution and, mothers who gave their last childbirth with assisted mode of delivery were 2.36 times more likely to feed their children with optimum dietary diversity. Women who have received PNC following their last child birth were 2.07 times more likely to feed their children with optimum dietary diversity. Mothers’ who went to health center for two hours and above, were 3.11 times more likely to feed their children with optimum dietary diversity. Women who fed their children four times and more per day were about three times more likely to feed their children with optimum dietary diversity (Table 4).

**Table 4:** The bivariate and multivariable logistic regression analysis showing factors associated with dietary diversity among children aged 6–23 months in Sinan *Woreda*, Northwest Ethiopia

Variables		Optimal DD	Sub-optimal DD	Crude Odds Ratio (COR)(95% CI)	AOR (95% CI)
Availability of media sources	Yes	39	116	3.22(2.04–5.08)***	2.77(1.64–4.68)***
	No	55	526	1	1
Cow milk available at house	Yes	22	76	2.28(1.33–3.88)***	2.38(1.31–4.35)***
	No	72	566	1	1
Mother involvement in decision making	Yes	82	522	1.57(0.83–2.97)	2.07(1.02–4.20)*
	No	12	120	1	1
Place of delivery	Health institution	75	321	4.59(2.58–8.18)***	2.40(1.24–4.67)**
	Health post	4	26	3.03(.94–9.78)	1.90(0.52–6.95)
	Home	15	295	1	1
Mode of delivery	Assisted delivery	15	28	4.16(2.16–8.13)***	2.36(1.12–4.98)*
	SVD	79	614	1	1
PNC follow up	Yes	70	270	4.02(2.46–6.55)***	2.07(1.18–3.62)**
	No	24	372	1	1
Knowledge of when to start CF	Yes	92	587	4.31(1.03–17.97)	3.07(0.67–14.00)
	No	2	55	1	1
Time to reach nearby health centers	≥2 h	21	74	2.21(1.28–3.79)***	3.11(1.66–5.83)***
	< 2 h	73	568	1	1
Meal frequencies of child	≥4 times/day	50	226	3.43(1.69–6.97)***	3.31(1.53–7.18)**
	3 times/day	34	261	2.02(0.97–4.20)	2.00(0.91–4.41)
	1-2times/day	10	155	1	1

Significant at *p-value < 0.05, **p-value < 0.01, and ***p-value < 0.001

## Discussion

This study assessed the factors associated with dietary diversity for children aged 6–23 months in Sinan *Woreda*. It identified that only 12.8% of the children received four or more food groups with in the 24 h preceding the survey. It is consistent with the findings from the studies done in Dangila 12.6% [28] and Tigiray 17.8% [30] districts of Ethiopia, India 13% [19] and Uganda 17.8% [31]. However, the finding is higher than the EDHS 2011 report [15] and lower than the studies done in India 27.4%, Tobago 48.23%, Cambodia 44%, Kenya 39.2%, 27.7%, Kamba district of Ethiopia 23.3% [22, 26, 29, 31–33]. The difference might occur due to time of study, socio-economic difference and geographical variation. The possible reasons for low dietary diversity practices in the study area could be low practice giving complimentary feeds after six months and the habit of the family (i.e. Preparing the family food together, no food preparation for children alone). Also low affordability of foods that is not available at home and those foods that are coasty are sold from the house instead of feeding their child. In addition, due to low feeding practices, animal source foods commonly used during holidays and ceremony other than usual.

The dominant food groups given to the children were grains, tubers and roots (98.8%) and legume (83.2%).This is comparable with the results from the studies done in Ethiopia [26, 28], and South Africa [34]. Feeding of flesh, egg, dairy products, vitamin rich fruits and vegetables and other fruits was low. This was in line with the studies done in Ethiopia [16, 26, 28], Central America [34], and Kenya [20]. The possible reason might be due to the unavailability of these food sources at household level; in which cereals, grains and tubers are the commonest products in the study area. People commonly consume those food items they cultivated and accessed from the market in low cost [35, 36].

The likelihood of feeding children with optimum dietary diversity was more among mothers who had media sources like radio and television (TV). Similar findings have been reported by the previous studies done in Northwest Ethiopia [28] and India [19]. We can understand from this result that availability of media sources at household might help the mothers getting information on recommended child feeding options [36].

Mothers who had available cow’s milk at household level were more likely to feed diversified food group than those who had not. Presence of accessible animal source foods at households helped women to feed their children with dairy products [36]. Similar result has been reported by the study conducted in southern Ethiopia Kemba district [26]. The possible reason might be the household that have cow’s milk, feed their child milk and milk products in addition to usual diet. Those mothers who were participating in decision making at household were more likely practicing optimal dietary diversity feeding to their children. The study done in Northwest part of Ethiopia had also revealed that feeding of optimum dietary diversity to their children is more among mothers who had involved in household decision making [28].

Among health utilization factors, those women who delivered their last child at health institutions were more likely to feed their children with diversified food. Similarly, those mothers who received postnatal care were more likely to feed their children with diversified food. This was in line with the study done in southern Ethiopia that showed the odds of feeding of diversified food was higher among mothers who received institutional delivery and postnatal follow up [26]. A study done in East African Regions [14] also revealed those hospital-based births shown to have a positive relationship with complementary food diversity. Health professionals might provide health education and counseling on dietary diversity when they deliver at health institutions and during postnatal care follow up visits [37]. Those who delivered their last child with assisted mode of delivery were more likely to feed diversified diet to their child than those who had spontaneous vaginal delivery. Mothers who gave birth through assisted mode of delivery might have prolonged stay at health institutions and get the opportunity to receive health education on infant and child feeding by senior providers [37].

Surprisingly, mothers residing relatively far from the health centers had higher odds of feeding their child with diversified diet. The possible reason might be due to frequent visit by health extension workers (HEWs). In rural Ethiopia, HEWs are working at health posts relatively far to health centers but closest to the community. They are responsible to provide health care services summarized into 16 packages at household level in the community [38, 39]. In addition, currently most of the child health activities are carried out at health post in Ethiopia. Children, whose meal frequency was four and more times per day, had higher odds of dietary diversity, This finding was in line with the study conducted in East African regions [14].

## Conclusion

Optimum dietary diversity among children aged 6–23 months in Sinan *Woreda* is low. Availability of media sources, availability of cow’s milk in the household, delivered at health institutions, assisted delivery, postnatal care, relatively far distance to reach to nearby health centers and feeding frequency four and above times/day was significant factors associated with optimal dietary diversity feeding of children aged 6–23 months. Infant and young child feeding practice according to the guideline should be implemented at community level and Mothers should be educated about how to prepare the diversified diet from locale available food groups for complimentary after 6 months. This study concluded that women involvement at household decision making improves dietary diversity of children. Ensuring maternal health service utilization can contribute for better dietary diversity of children aged 6–23 months. The government should strengthen the maternal health service utilization by expanding institutional delivery service, PNC service and counseling on child feeding. Information and advertising about IYCF are disseminated to grass-root level. Large scale an interventional based research has to be conducted.

## Abbreviations

ANC: Ante Natal Care
AOR: Adjusted Odds Ratio
CF: Complementary Feeding
CHIS: Community Health Information System
COR: Crude Odds Ratio
DDS: Dietary Diversity Score
EDHS: Ethiopian Demographic Health Survey
HEWs: Health Extension Workers
IYCF: Infant and Young Child Feeding
PNC: Post Natal Care
SDG: Sustainable Development Goals
WHO: World health organization
